# By-product distribution and cytotoxicity assessment of ZnO-assisted photocatalytic degradation of reactive blue 250 dye

**DOI:** 10.1016/j.heliyon.2024.e39670

**Published:** 2024-10-23

**Authors:** Tanveer Hussain Bokhari, Aniqa Naveed, Muhammad Kaleem Khosa, Atta ul Haq, Majid Muneer, Mazhar Iqbal, Osama A. Mohammed, Ahmed S. Doghish, Mustafa Ahmed Abdel-Reheim, Munawar Iqbal, Arif Nazir

**Affiliations:** a Department of Chemistry, Government College University, Faisalabad, 38000, Pakistan; b National Institute for Biotechnology and Genetic Engineering, Faisalabad, Pakistan; c Department of Pharmacology, College of Medicine, University of Bisha, Bisha, 61922, Saudi Arabia; d Department of Biochemistry, Faculty of Pharmacy, Badr University in Cairo (BUC), Badr City, Cairo, 11829, Egypt; e Biochemistry and Molecular Biology Department, Faculty of Pharmacy (Boys), Al-Azhar University, Nasr City 11231, Cairo, Egypt; f Department of Pharmacology, College of Pharmacy, Shaqra University, Shaqra 11961, Saudi Arabia; g Department of Pharmacology and Toxicology, Faculty of Pharmacy, Beni-Suef University, Beni Suef 62521, Egypt; h School of Chemistry, University of the Punjab, Lahore 54590, Pakistan; i Department of Chemistry, The University of Lahore, Lahore, Pakistan

**Keywords:** Photo-catalysis, Dyes, Advanced oxidation processes, Decolorization, Nanoparticles

## Abstract

This research examined the effectiveness and feasibility of utilizing ultraviolet (UV) assisted photo-catalysis to treat wastewater effluents from textile production containing reactive blue 250 (RB 250) dye. Molecular oxygen and active species like O_2_^•−^, HO_2_^•^, H_2_O_2_ and ^•^OH play crucial roles in the degradation process. Additionally, the degradation of dyes is influenced by several factors, including dye concentration, duration of UV irradiation, pH levels, concentration of H_2_O_2_, and the catalyst. The concentration of H_2_O_2_ and catalyst dose for the decolorization was studied at 0.6 mL and 0.5 g respectively. The discoloration was higher at low dye concentration, high H_2_O_2_ concentration, acidic conditions and high catalyst concentration. The maximum degradation (97 %) of RB 250 dye was obtained in the presence of zinc oxide nanoparticles within 90 min. The extent of decolorization of the dye was determined by UV–Vis spectroscopy. Fourier transform infrared spectroscopy (FTIR) was employed to analyze the changes in functionalities after degradation. The disappearance of characteristic peaks associated with specific groups within the dye molecule confirmed the extensive degradation of RB 250 dye. LCMS analysis was conducted to examine the intermediates and a mechanistic degradation pathway was subsequently proposed. The cytotoxicity of the irradiated dye samples was evaluated through a hemolytic test both pre and post-treatment. The findings suggest that the UV/H_2_O_2_/ZnO treatment represents a promising approach for effectively degrading RB 250 dye.

## 1 Introduction

Currently, organic colorants are regarded as the primary pollutants in the effluents produced by a wide range of factories, particularly the manufacturing industries of textiles, paper, watercolor, ink, rubber-based materials and plastics. According to estimates, fifteen to twenty percent of the dyes are lost during manufacture or treatment and released in wastewater from industry [[1], [2], [3], [4]]. Organic colorants are typically found in the effluents in quantities that vary between 5 and 1500 mg/L, which poses major risks to the surroundings and the lives of living things, including the development of cancer and mutagenesis consequences [[5], [6], [7]].

Although dyes are resistant to sunlight as well as oxidizing agents [8], traditional approaches for removing dyes from effluents such as biological procedures, flocculation, filtration, precipitation, and coagulation have several limitations [[9], [10], [11]], mainly because of the poor elimination effectiveness. The adsorption onto carbonaceous substances, such as nanotubes made from carbon and activated carbon, was regarded as an effective dye elimination method amongst physical techniques [[12], [13], [14]], whereas enzymatic breakdown was regarded as an effective dye elimination approach amongst biological procedures [15,16]. When using chemical methods to treat diverse organic dyes under UV light, heterogeneous photocatalytic oxidation (HPO) has been suggested as an effective choice [[17], [18], [19], [20]].

Reactive dyes exhibit a strong affinity for forming covalent bonds with –OH, –NH, or –SH groups found in textile fibers composed of cotton, wool, silk and nylon. Their desirable characteristics, including vibrant color, exceptional colorfastness, and easy application establish reactive dyes as the most practical class of dyes, particularly in textile and dyeing industries [6,[21], [22], [23], [24]]. The issue of highly pigmented effluents containing these dye pollutants arises from the hydrolysis of reactive groups such as anthraquinone, azo, and phthalocyanine during the dyeing process [25,26].

Reactive blue 250 dye is a pollutant that poses a problem due to its water solubility and difficulty in degrading, just as various dyes used in the textile sector [27]. If it exists in reservoirs, it may affect the colour of the water, which may obstruct daylight from entering aquatic life and hinder the photosynthesis and development of aquatic creatures [28]. Batch testing was employed for conducting decomposition experiments, evaluating crucial procedural factors such as catalyst loading, dye quantity, H_2_O_2_ concentration, initial solution pH, and catalytic persistence and reusability. Consequently, these factors present notable obstacles for degradation via commonly employed treatment methods like adsorption, coagulation, filtration, precipitation, and ion exchange [29,30].

Physicochemical methods also come with inherent limitations. Physical techniques frequently give rise to secondary pollution issues. Chemical treatments utilizing potent oxidants like chlorine or ozone prove effective against dye pollutants but are both economically and environmentally detrimental. The generation of toxic intermediates during chemical treatment processes poses hazards to both human health and aquatic ecosystems. Moreover, highly resilient synthetic dyes or organic compounds impede the effective functioning of biological methods [31].

The advanced oxidation processes (AOPs) possess the benefits above other methods in that they generate innocuous substances like carbon dioxide, water, and mineral acids. Additionally, the AOPs do not release any additional contaminants [32,33]. Scientists and researchers have been concentrating on the AOPs to get the best and most practical outcomes for water purification utilizing metal oxide semiconductors. Zinc oxide [34] is one of the most widely employed metal oxide semiconductors in photocatalytic processes.

ZnO serves as an n-type semiconductor that generates excitonic, optically induced ultraviolet (390 nm) laser activity and has a large bandwidth of 3.36 eV. Zinc oxide possesses excellent thermal characteristics, high resistance to damage by ionizing radiation, excellent transparency, stability in structure, biological reliability, as well as efficient ultraviolet range absorption of light [35]. Zinc oxide, therefore, offers a broad range of possible uses, comprising photo-catalysis, antimicrobial products, solar energy cells, and optical electronics. Zinc oxide has powerful redox characteristics, superior biological compatibility, and broad bandgap. Zinc oxide responds to only five percent of the ultraviolet radiation within the sunlight's spectrum as well as 0.1 percent of the ultraviolet (UV) rays in internal exposure [[36], [37], [38], [39]]. The inclusion of transition metal ions is a useful tool for achieving the necessary features and making mirror adjustments to the chemical and physical structure at the nanoscale [40].

The primary aim of the present study is to synthesize ZnO nanoparticles via the sol-gel method. The synthesized nanoparticles underwent characterization using UV–Vis, FTIR, and XRD analysis. The research also involved examining the impact of initial dye concentration, pH, and initial dosage of zinc oxide catalyst on the degradation of RB 250 dye.

## 2 Materials and methods

The chemicals used in this study were of analytical grade and obtained from Sigma-Aldrich. Throughout the study, distilled water was used to prepare the necessary experimental solutions.

**Fig. 1:**
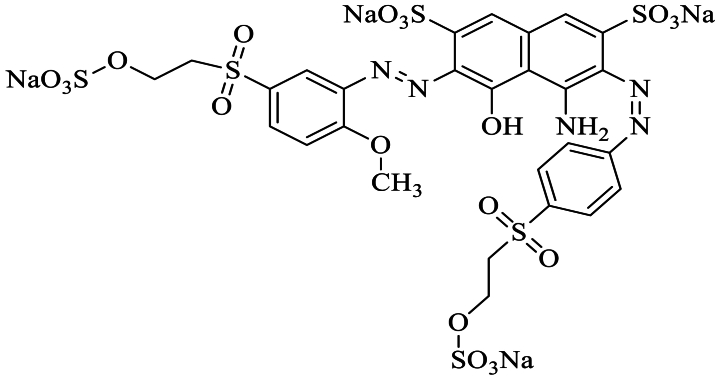
Structure of reactive blue 250 dye showing different functionalities.

UV source at 254 nm, 144 W was used to provide radiations to different samples of RB 250 dye, while a gamma radiation source (Cs-137) was used. The UV–Vis double-beam spectrophotometer (STA-8200) and FTIR spectrometer (U-2001, Shimadzu, Japan) were utilized for observing the spectra of various samples. The dye (Fig. 1; Table 1) solution was introduced into the sample cell, and subsequently, the spectrophotometer was scanned across the entire visible range (780-380 nm) of light.

**Table 1:** Physiochemical features of the RB 250 dye.

**Dye**	**Reactive blue 250**
CAS No.	93951-21-4
Mol. formula	C_27_H_23_N_5_Na_4_O_20_S_6_
Mol. mass	1020.89
Chemical nature	Anionic blue 250
Chromophore group	Azo group
Color index name	Reactive Blue 250
*λ*_max_(nm)	604
Reactive group	Vinyl sulfone group

### 2.1 Experimental procedure

Batch tests for the photocatalytic decomposition of RB 250 dye were carried out using a 254 nm ultraviolet mercury lamp. The reactor was originally equipped with 100 mL of RB 250 aqueous solution for each successful experiment and stirring was maintained using a magnetic stirrer. In this work, to prepare a 1000 ppm stock solution, 1 g dye powder was dissolved in 1000 mL distilled water, and then the desired concentrations were prepared. In UV/H_2_O_2_, different concentrations (0.2–0.9 mL) of H_2_O_2_ were used in different dye solutions and irradiated with UV light. If ultraviolet/H_2_O_2_/ZnO was used, then 0.6 mL H_2_O_2_ was initially added into the dye solution, after which the pH 2 was adjusted using 1M hydrochloric acid or 1M sodium hydroxide. Then 0.1 g of ZnO NPs was added to the above dye solution followed by vigorous stirring for 1 h in a dark environment to promote equilibrium between the adsorption and desorption processes. Various dye-catalyst combinations were irradiated using a 254 nm ultraviolet mercury lamp with 144 W power. The samples were stirred while under illumination. After 30 min about 3 mL of each sample were drawn utilizing a syringe and the drawn sample was examined by UV/Vis spectrophotometer. Reactive blue 250 dye shows maximum absorbance at 604 nm. For each dye, the dose of ZnO nanoparticles was changed from 0.1 g to 0.7 g to study the impact of catalyst concentration on the process of degradation. The concentration of RB 250 was found out from the absorbance value using the pre-established calibration curve.

### 2.2 Toxicity assessment

The cytotoxicity parameter was assessed in both untreated and treated samples of reactive blue 250 dye. So, the cytotoxicity of RB 250 dye was tested by performing hemolytic assays on human RBCs.

Freshly drawn human blood (3 mL) was deposited in heparinized tubes, thoroughly mixed and then emptied into a sterile 15 mL falcon tube which was then centrifuged for 5 min to stop coagulation. The supernatant was removed and the RBCs were then washed three times in sterile, isotonic, PBS solution (5 mL) which was chilled to 4^o^C and had a pH of 7.4. After being cleaned, the RBCs were suspended in 20 mL of cold PBS along with the addition of diluted blood cell suspension, 60 mg/L of RB 250 dye solution was added to 2 mL Eppendorf tubes. The samples underwent a 35min incubation period at 37^o^C. Tubes were incubated and stirred for 10 min, then put on ice for 5 min before being centrifuged for at least 5 min. After centrifugation, the supernatant (100 L) was removed from the tubes and diluted with chilled PBS (900 L). All Eppendorf's were kept on ice after diluting. The Eppendorf (200 L) mixture was then transferred to individual 96 well plates. Each experiment employed phosphate buffer saline (PBS) as a negative control and 0.1 % Triton X-100 as a positive control [41].

### 2.3 Fourier transform infrared spectroscopy

Using the technique of Fourier transform infrared spectroscopy (FTIR) assessment, the characteristics of the dye and treated samples were examined. The characterization includes analyzing dry dye powder as well as deteriorated dye samples that were verified utilizing a (U-2001, Shimadzu, Kyoto, Japan) spectrophotometer. The frequency span of the recorded spectra (128 scans at a resolution of 2 cm^−1^) was 4000-400 cm^−1^, with a resolution enhancement factor of 1.5 and a bandwidth of 15 cm^−1^.

### 2.4 Liquid chromatography-mass spectrometry

By using LC-MS analysis, the dye's breakdown procedure was examined. Through the quasi-molecular ion (MH^+^), the LC-MS spectra reveal the molecule's mass. The invention of LC separation and MS coupling represents a significant advancement in our ability to study the emergence and disintegration of chemical intermediates. This coupling method may also be used to differentiate between and divide polar molecules without derivatizing them.

## 3 Results and discussion

### 3.1 Degradation of dye solution

**Fig. 2:**
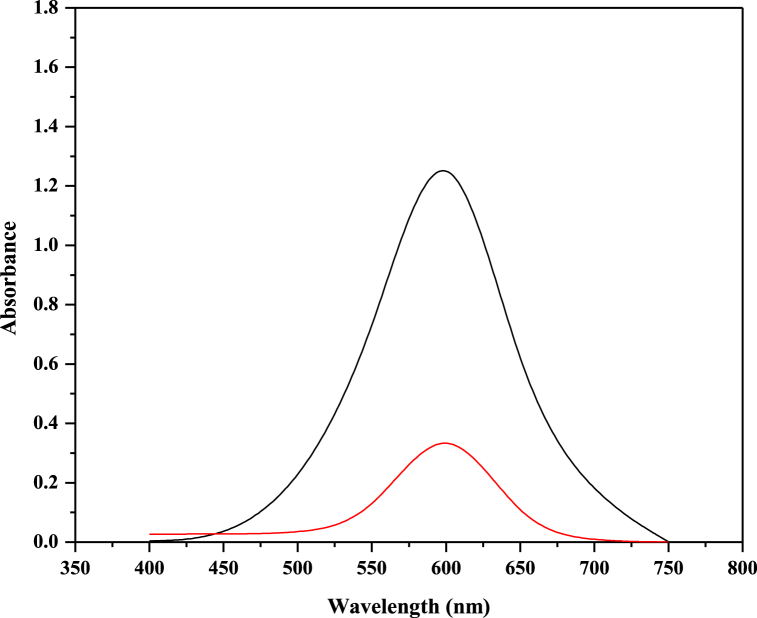
UV–Vis spectrum of RB 250 before and after UV irradiation.

**Fig. 3:**
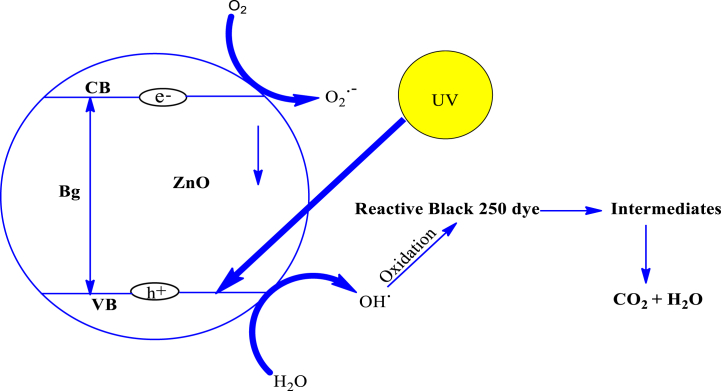
Mechanism of photocatalytic degradation of RB 250 using ZnO as a catalyst [44].

The peak in the UV–Vis spectra of reactive blue 250 is at 604 nm (Fig. 2). The absorption peak at 604 nm was declined and almost vanished in the deterioration process during the duration of the investigation. This indicates that the chromophore system was being demolished. The *λ*_max_ (604 nm) of the dye was selected for further degradation experiments. While Fig. 3 shows the mechanism behind photo-catalysis.

### 3.2 Photocatalytic degradation mechanism of reactive blue 250 dye

**Fig. 4:**
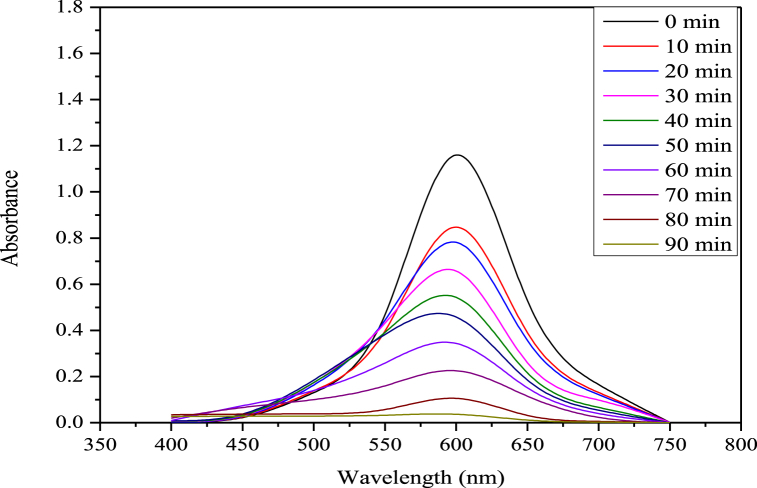
UV–vis absorbance spectra of RB 250 as a function of time over ZnO nanoparticles under UV light irradiation.

Zinc oxide nanoparticles were used to photo-catalytically degrade the RB250 while being exposed to ultraviolet light from a 144 W UV illuminator. Fig. 4 displays the ultraviolet (UV)-visible spectrum of RB250 deterioration by ZnO nanoparticles during the time range of 0–90 min when UV light is present.

**Fig. 5:**
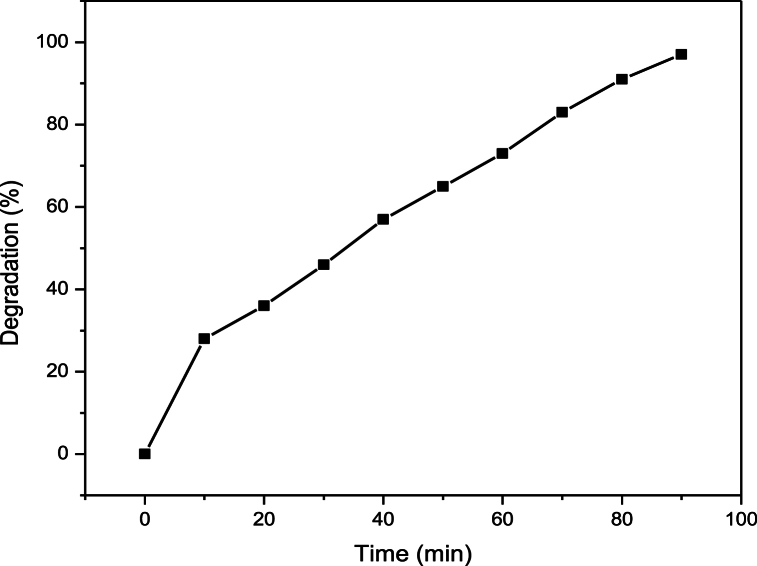
Calibration curve of the degradation rate (%) versus time interval.

Fig. 4 demonstrates that as the UV exposure period is increased, the absorbance of RB250 is reduced, indicating the decomposition of RB250 with zinc oxide nanoparticles. After 90 min, the absorbance virtually reaches zero, indicating that the RB 250 has been completely degraded. The calibration curve of the deterioration rate (%) versus time interval is shown in Fig. 5. The first rapid increase in the %age breakdown of RB 250 is presumably caused by the adhering of dye to the surface of ZnO NPs. After 90 min, a quick breakdown of RB 250 was attained with a 97 % degradation rate.

The degradation reaction mechanism illustrated that the sample is exposed to ultraviolet radiations of photon energy larger than the bandgap energy of zinc oxide. Zinc oxide nanoparticles are photo-induced to produce electron-hole pairs as described in eq. (1), when exposed to ultraviolet radiation with photon energies larger than the bandgap energy of zinc oxide.

(1) ZnO + hv → (e^−^_CB_) + (h ^+^ _VB_)

According to equations (2), (3), (4), the electron-hole pairs are created by photoexcitation which is proceeded through redox processes. In a sequence of oxidation processes, the holes (h^+^ions) liberate hydroxide ions, which then transform into hydroxyl radicals (eqs. (2), (3))). In equation (4), electrons convert oxygen to produce a superoxide radical. In equations (5), (6), the superoxide radical reacts with H^+^ ions to generate various hydroperoxyl molecules which combine to produce hydrogen peroxide [42].

(2) H_2_O + (h ^+^ _VB_) → ^−^OH + H^+^

(3) ^−^OH + (h + _VB_) → ^•^OH

(4) O_2_ + (e^−^_CB_) _→_ O_2_^−^

(5) H^+^ + O_2_^−^ → HO_2_^•^

(6) HO_2_^•^ + HO_2_^•^^→^ O_2_ + H_2_O_2_

Hydroxyl radicals are produced as a consequence of further interaction between H_2_O_2_ and superoxide radicals, as described in eq. (7). As seen in eq. (8), excessive H_2_O_2_ is also converted into hydroxyl radicals under the influence of light.

(7) O_2_^−^ + H_2_O_2_ → ^−^OH + ^•^OH + O_2_

(8) hv + H_2_O_2_ → 2^•^OH

According to eqs. (9), (10), the principle oxidizing agents are the liberated hydroxyl radicals that dissolve complex organic contaminants which are adsorbed on ZnO and transform them into various intermediates and green products like H_2_O and CO_2_ [43].

(9) Organic pollutants → ^•^OH + Intermediates

(10) Intermediates → CO_2_ + H_2_O

As a result, when exposed to light, ZnO NPs produce pairs of electrons and holes that move to the surface and start the reactions. On the surface of the catalyst, holes join with hydroxyl groups to form hydroxyl radicals, while electrons mix with oxygen to form superoxide radicals. The primary species are the radicals that convert reactive blue 250 into eco-friendly byproducts such as CO_2_, H_2_O, and mineral acids.

### 3.3 Effect of dye concentration

**Fig. 6:**
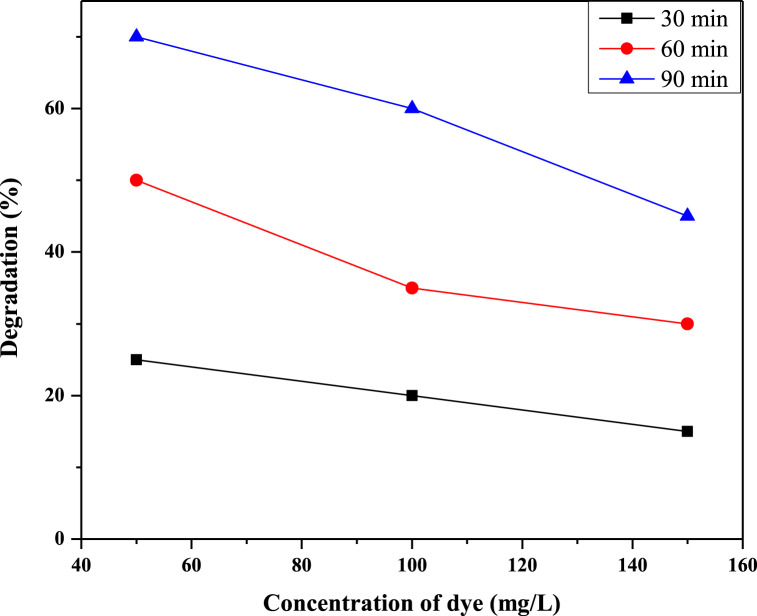
Effect of RB250 concentration on the degradation of RB250.

The effect of the initial concentration of RB 250 on degradation (%) was investigated by changing the concentration from 50 to 150 ppm while maintaining other parameters. The %age of degradation was observed to reduce when the concentration of RB 250 was increased, as shown in Fig. 6. The result indicated that the degradation rate was reduced from 70 % to 45 % when the RB 250 concentration was increased from 50 ppm to 150 ppm after 90 min of exposure to UV radiations. When the concentration of RB 250 increases, the number of active sites decreases. When the concentration of RB 250 increases, fewer photons reach the catalyst surface, and the production of OH radicals declines. Photodegradation is impossible due to increases in the concentration of RB 250 because the surface area decreases [45].

### 3.4 Effect of UV irradiation time

**Fig. 7:**
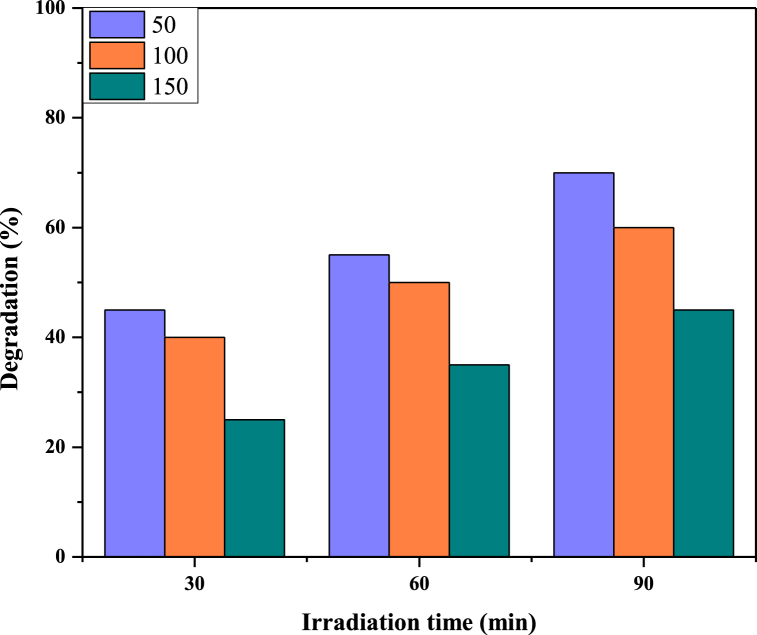
Effect of time on the degradation of RB250.

**Fig. 8:**
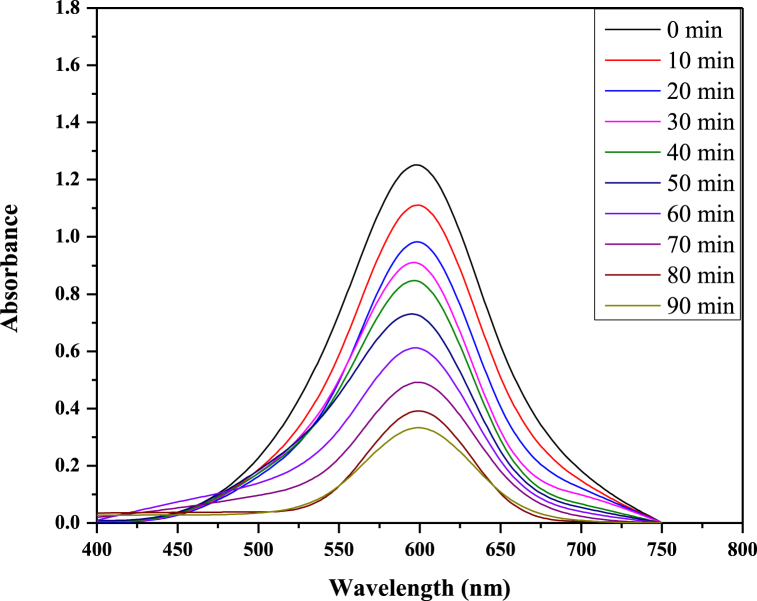
UV–Vis absorbance spectra of RB250 as a function of time under UV light irradiation.

Reactive blue 250 solutions at different concentrations (50 mg/L, 100 mg/L, and 150 mg/L) were exposed to UV radiation within a reactor for durations spanning 30–90 min. The pH of the sample solutions was adjusted to an optimized value of 2. It has been observed that absorbance decreased as time of exposure to UV light increased. Hence it is attributed to an increased degradation rate. After 30 min of UV irradiation, 45 % degradation was observed for 50 mg/L solutions of reactive blue 250. After 90 min, 70 % degradation was observed under UV irradiations alone. Hence by increasing UV exposure time, degradation efficiency increased. Fig. 7 shows the percentage degradation of reactive blue 250 at different UV irradiation times. Fig. 8 shows that as the time increased, the rate of degradation was also increased.

### 3.5 Effect of H_2_O_2_ concentration

Different concentrations of H_2_O_2_ (0.2 mL, 0.4 mL, 0.6 mL, 0.8 mL) were added to RB 250 solutions and were irradiated with UV light. Other parameters were kept constant. It has been observed that UV/H_2_O_2_ speeds up the photodegradation process in comparison to UV treatment only. Reactive Blue 250 degradation was increased by increasing concentrations of H_2_O_2_ up to a certain limit. At the concentration of 0.2 mL, the percentage degradation of RB 250 was 69 % for 50 ppm and when H_2_O_2_ concentration was 0.6 mL, degradation was 85 % for 50 ppm. Also, degradation was highest (85 %) at 0.6 mL of H_2_O_2_ hence it was considered an optimum condition. More increase in H_2_O_2_ amount causes a decrease of degradation percentage such as in this case degradation percentage was 78 % at 0.8 mL of H_2_O_2_. This is attributed to the scavenging effect of hydrogen peroxide. The reason is that at higher hydrogen peroxide concentrations, ^•^OH radical and H_2_O_2_ react to produce HO_2_^•^ radical and these radicals do not take part in the degradation process of RB250.

^•^OH + H_2_O_2_ → H_2_O + HO_2_^•^

^•^OH + HO_2_^•^ → O_2_ + H_2_O

**Fig. 9:**
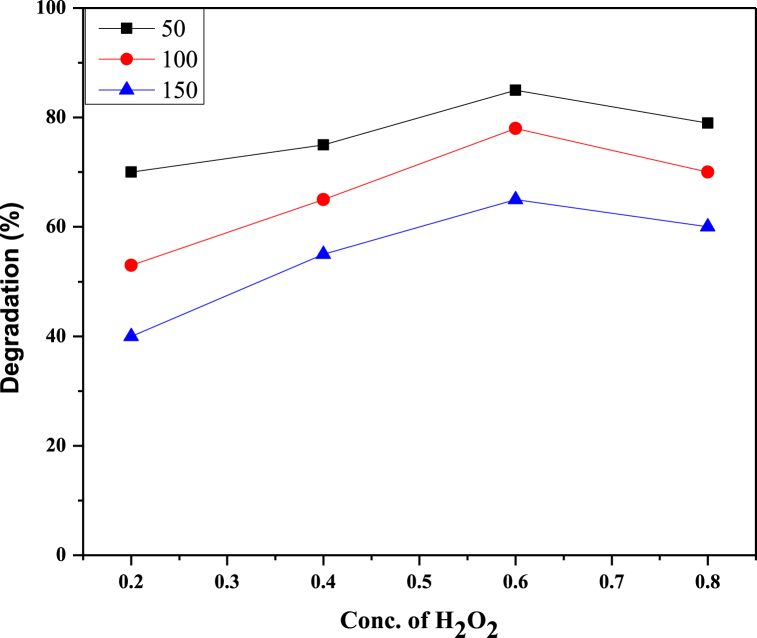
Effect of H_2_O_2_ concentration on RB250 degradation.

Optimizing the H_2_O_2_ is essential for enhancing the effectiveness of the UV/H_2_O_2_ process while reducing the risk of side effects and treatment expense [46]. The effect of hydrogen peroxide can be seen in Fig. 9 and it indicated that in the above optimum concentration of H_2_O_2_, the percentage degradation of RB 250 is decreased.

### 3.6 Effect of pH

**Fig. 10:**
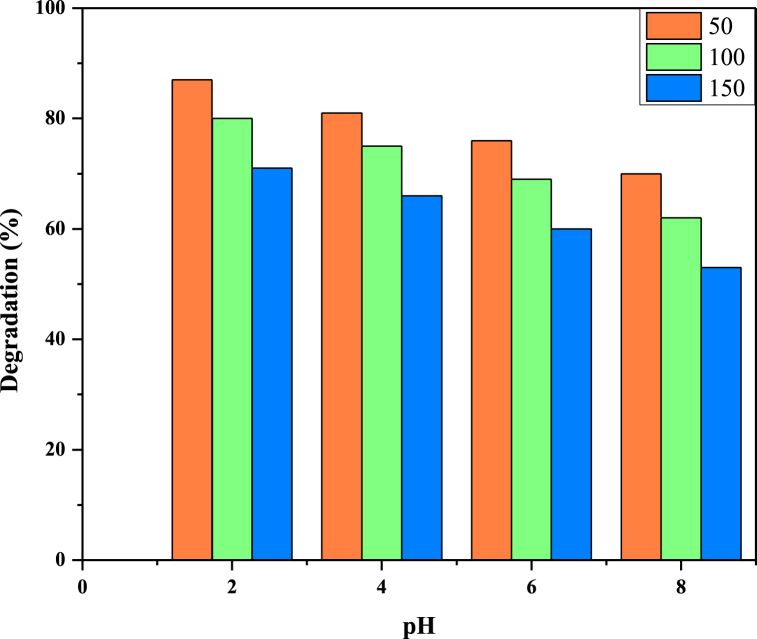
Effect of pH on RB250 degradation.

The effect of pH on the degradation of reactive blue 250 was evaluated by changing the pH from 2 to 8 at RB 250 concentrations of 50 ppm, 100 ppm and 150 ppm and 0.6 mL of H_2_O_2_ as given in Fig. 10. The results were indicated that degradation of reactive blue 250 was greater in acidic pH (pH = 2 & 4) than basic pH (pH = 4) especially pH 2 was more favorable to the photodegradation process because azo dyes are quickly adsorbed on the catalysts in an acidic condition (pH = 2). At pH 2, 87 % reactive blue 250 degradation was observed, which decreases as the pH increases, and the RB 250 degradation was 70 % in the basic medium (pH = 8) [47].

### 3.7 Effect of ZnO

**Fig. 11:**
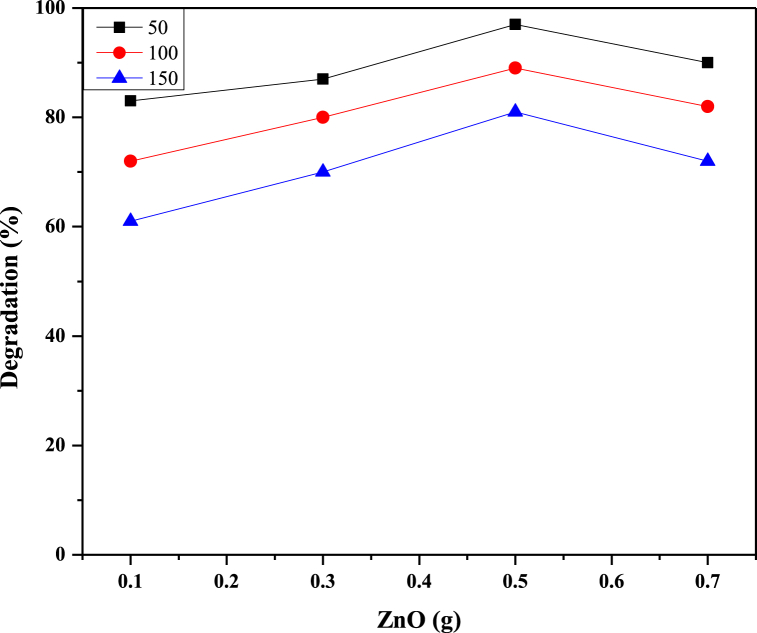
Effect of ZnO concentration on RB250 degradation.

It can be noted from Fig. 11 that the efficiency of degradation was increased from 82 % to 97 % for ZnO with the catalytic dosage ranging from 0.1 to 0.5 g by keeping all parameters constant. When the catalytic dose increases, the percentage degradation of RB 250 increases due to additional catalytic activating sites and increased electron excitation rate in conjunction with UV, producing more hydroxyl radicals to degrade the RB 250 [48]. Then increasing the catalyst dosage from 0.5 g has no noticeable effect on the rate of degradation. It can be explained in terms of a decrease in the penetration of UV light due to the increased effect of scattering and turbidity of the suspension [49].

### 3.8 FTIR analysis

**Fig. 12:**
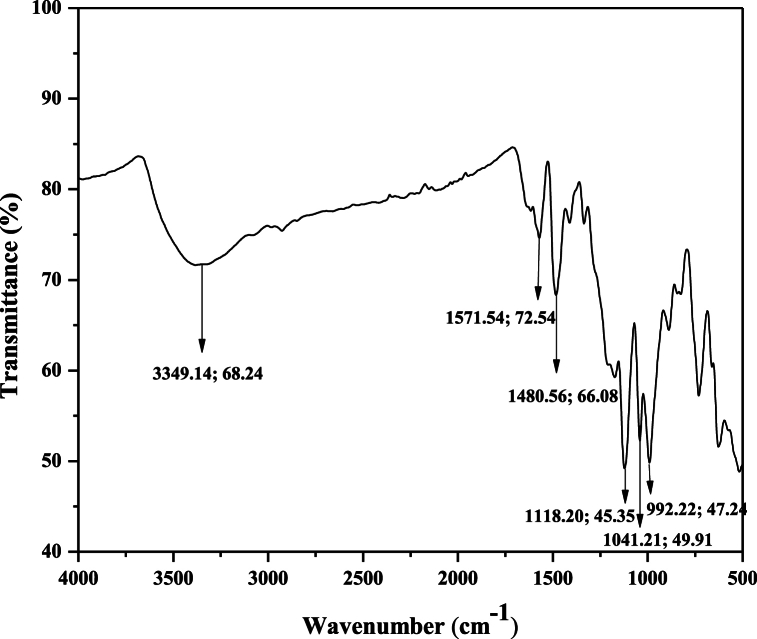
FTIR spectrum of RB 250 before irradiation shows the presence of different functional groups.

**Fig. 13:**
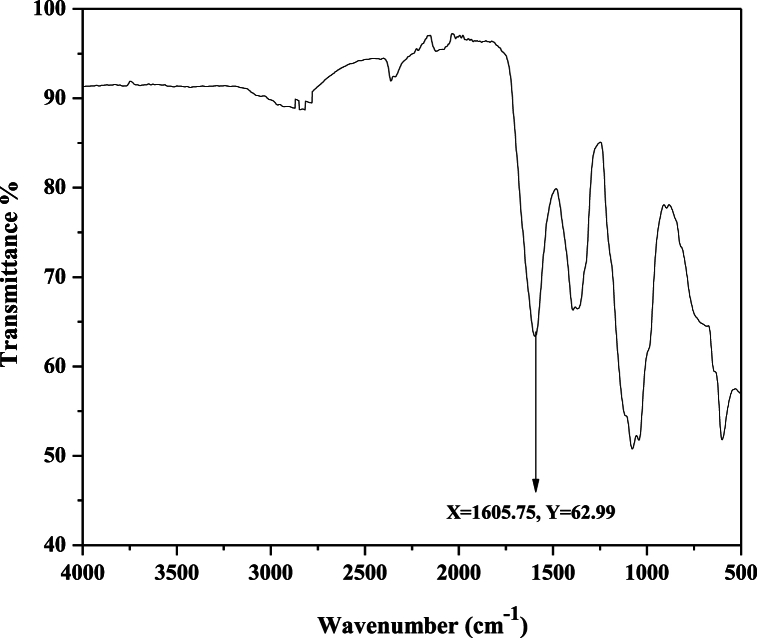
FTIR spectrum of RB 250 after irradiation shows the presence of different functional groups.

FTIR spectroscopy was employed to identify the diverse functional groups present. Fig. 12 displays the FTIR spectra of reactive blue 250 dye before irradiation, while Fig. 13 presents the spectra after irradiation. The results of the FTIR spectrum indicated the wide peak at 3349 cm^−1^ which represented the OH group. The main sharp peaks at 1571 cm^−1^, 1480 cm^−1^, 1118 cm^−1^, 1041 cm^−1^ and 992 cm^−1^ belonged to N=N azo group, aromatic group, C-O group, S=O group and C-H bending respectively. The FTIR graph after treatment shows a peak at 1605 cm^−1^ which indicates the elimination of the other group as a result of the degradation of RB 250 dye. The FTIR spectrum of reactive blue 250 dye indicated that it had been degraded as a consequence of the destruction of chromophores by UV radiation.

### 3.9 Toxicity study of reactive blue 250 dye

**Fig. 14:**
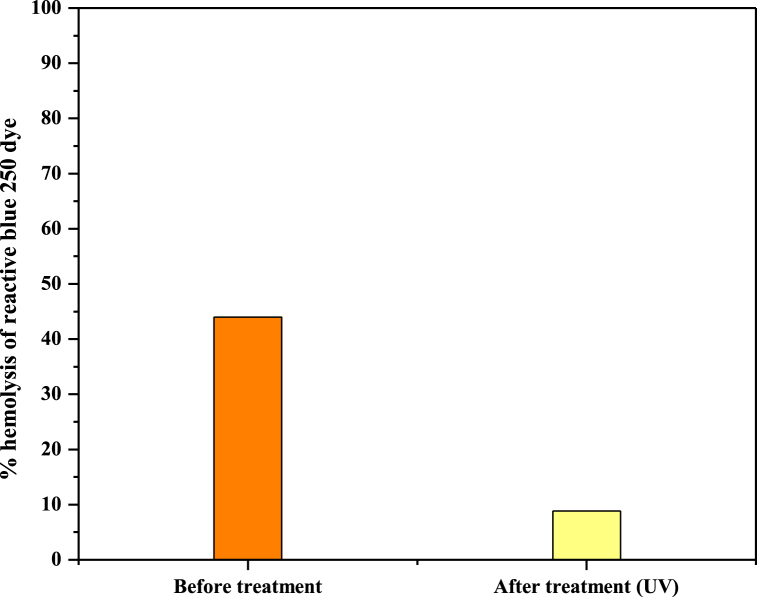
Percentage hemolysis of reactive blue 250 samples before and after irradiation. (For interpretation of the references to color in this figure legend, the reader is referred to the Web version of this article.)

Results demonstrate the cytotoxicity studies of an un-irradiated sample of reactive blue 250 (50 mg/L, 0.6 mL H_2_O_2_) that was 44 % and it was reduced to 8.84 % when irradiated with UV radiation. Percentage hemolysis of reactive blue 250 is shown in Fig. 14 and Table 2.

**Table 2:** The hemolytic test demonstrated a decrease in cytotoxicity following UV radiation treatment.

**Control**	**Sample**	**Absorbance**	**N.C Abs.**		**P. C Abs.**		**Hemolytic test (%)**
	Untreated	0.082	0.029	0.029	0.328	0.44	44
	Treated with UV radiations	0.058	0.029	0.029	0.328	0.088	8.84
0.1 % Triton X-100	Positive control	0.328	0.029	0.299	0.328	0.912	91.2
Phosphate Buffer Saline	Negative control	0.029	0.029	0	0.328	0	0

### 3.10 LCMS analysis

**Fig. 15:**
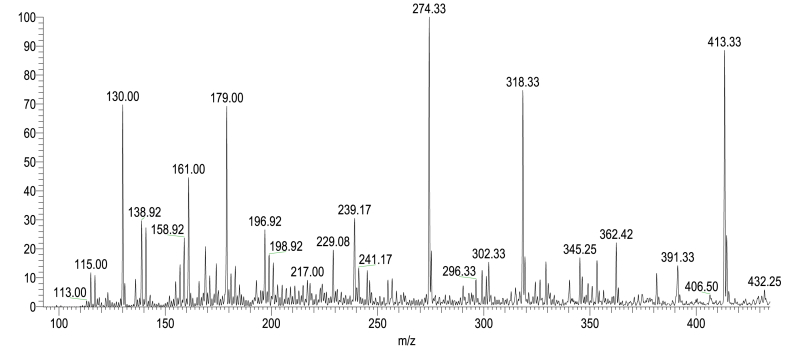
LCMS spectrum of reactive blue 250 dye for 50 ppm using 0.6 mL H_2_O_2_ and UV irradiation time 90 min. (For interpretation of the references to color in this figure legend, the reader is referred to the Web version of this article.)

**Fig. 16:**
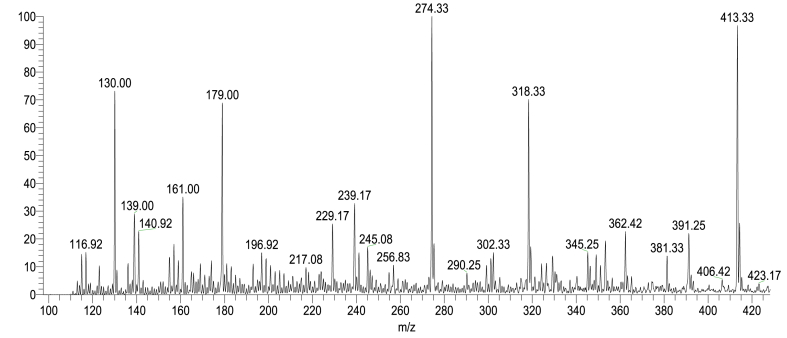
LCMS spectrum of reactive blue 250 dye for 50 ppm using ZnO 0.5 g, 0.6 mL H_2_O_2_, pH 2 and UV irradiation time 90 min. (For interpretation of the references to color in this figure legend, the reader is referred to the Web version of this article.)

**Fig. 17:**
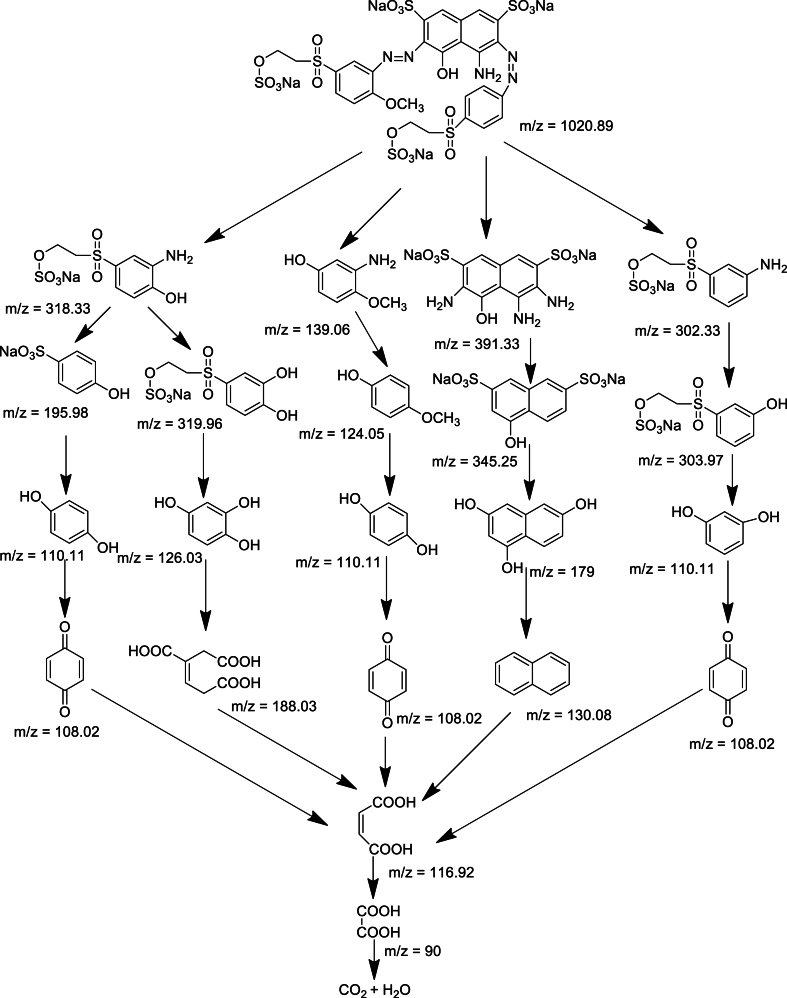
Proposed degradation pathway of reactive blue 250 dye.

LC-MS analyses (Fig. 15, Fig. 16; Table 3, Table 4) were performed using a linear ion trap mass spectrometer in positive mode with electrospray ionization for evaluating the degradation product of RB 250 by interpreting their mass spectra data, which displayed their molecular ion peaks concerning *m*/*z*. The mass-to-charge ratio (*m*/*z*) range for the degraded product ion was scanned from 50 to 500, the retention time was 0.01–0.42 min for all the samples. It involves different pathways (Fig. 17). The main species involved in the degradation process is hydroxyl radical (^•^OH) which attacks the C-C and C-N of the chromophore group and the C-S bond between the aromatic ring and the sulphonate group and lead to the formation of different degraded product with *m*/*z* value at 318.33, 139.06, 302.33, 345.25, 179, 130 and 116.92.

**Table 3:** Chemical structures of intermediates of RB 250 for 50 ppm using UV/H_2_O_2_ identified by LC/MS analysis.

**Sr. No.**	**Name of compounds**	**Structural formula**	**m/z value**	**Mol. Wt.**	**Status**
1.	sodium 2-((3-amino-4-hydroxyphenyl) sulfonyl) ethylsulphate		318.33	319.29	Detected
2.	3-amino-4-methoxyphenol		139.06	139.15	Detected
3.	sodium 2-((3-aminophenyl) sulfonyl) ethylsulphate		302.33	303.29	Detected
4.	naphthalene-1,3,6-triol		179	179.11	Detected
5.	Naphthalene		130	128.17	Detected
6.	maleic acid		116.92	117.09	Detected

**Table 4:** Chemical structures of intermediates of RB 250 for 50 ppm using ZnO identified by LC/MS analysis.

**Sr. No.**	**Name of compounds**	**Structural formula**	**m/z value**	**Mol. Wt.**	**Status**
1	sodium 2-((3-amino-4-hydroxyphenyl)sulfonyl)ethylsulphate		318.33	319.29	Detected
2	3-amino-4-methoxyphenol		139.06	139.15	Detected
3	sodium 2-((3-aminophenyl)sulfonyl) ethylsulphate		302.33	303.29	Detected
4	sodium 4-hydroxynaphthalene-2,7-disulphonate		345.25	345.98	Detected
5	naphthalene-1,3,6-triol		179	179.11	Detected
6	Naphthalene		130	128.17	Detected
7	maleic acid		116.92	117.08	Detected

## 4 Conclusion

The effectiveness and viability of UV-assisted photo-catalysis for degrading RB 250 dye was evaluated. Molecular oxygen and various active species, including O_2_^•^−, HO_2_^•^, H_2_O_2_, and ^•^OH, were found to play crucial roles in the degradation process, which occurs through a sequence of reactions. Optimum conditions for decolorization were determined to be 0.6 mL and 0.5 g for H_2_O_2_ and catalyst dose, respectively. Higher discoloration rates were observed at lower dye concentrations, higher H_2_O_2_ concentrations, acidic conditions, and increased catalyst concentrations. The presence of zinc oxide nanoparticles as a catalyst resulted in the maximum degradation (97 %) of RB 250 dye within 90 min. The extent of decolorization was assessed using UV–Vis spectroscopy, while FTIR was employed to analyze the products obtained after complete degradation. The removal of specific groups in the dye molecule confirmed the maximum degradation of RB 250 dye. LCMS analysis was utilized to scrutinize the intermediates, leading to the proposal of a mechanistic degradation pathway. Furthermore, the cytotoxicity of the irradiated dye samples was evaluated using a hemolytic test before and after treatment, indicating the potential effectiveness of the UV/H_2_O_2_/ZnO treatment method for degrading RB 250 dye. Overall, the findings suggest that UV/H_2_O_2_/ZnO treatment can serve as an effective approach for the degradation of RB 250 dye in textile wastewater effluent, highlighting its potential for addressing environmental concerns related to dye pollution.

## CRediT authorship contribution statement

**Tanveer Hussain Bokhari:** Supervision, Conceptualization. **Aniqa Naveed:** Writing – original draft, Methodology, Investigation. **Muhammad Kaleem Khosa:** Writing – original draft, Methodology, Investigation. **Atta ul Haq:** Validation, Software, Project administration. **Majid Muneer:** Validation, Software, Project administration. **Mazhar Iqbal:** Validation, Software, Project administration. **Osama A. Mohammed:** Validation, Software, Project administration. **Ahmed S. Doghish:** Visualization, Resources, Data curation. **Mustafa Ahmed Abdel-Reheim:** Visualization, Resources, Data curation. **Munawar Iqbal:** Visualization, Resources, Data curation. **Arif Nazir:** Writing – review & editing.

## Declaration of competing interest

The authors declare that they have no known competing financial interests or personal relationships that could have appeared to influence the work reported in this paper.

## Data Availability

Data will be made available on request.
